# Pyridine-4-carboxamidoxime *N*-oxide

**DOI:** 10.1107/S2414314620013358

**Published:** 2020-10-09

**Authors:** Clifford W. Padgett, Kirkland Sheriff, Will E. Lynch

**Affiliations:** a Georgia Southern University, 11935 Abercorn St., Department of Chemistry and Biochemistry, Savannah GA 31419, USA; Benemérita Universidad Autónoma de Puebla, México

**Keywords:** crystal structure, *N*-oxide, oxime, hydrogen bonding, supra­molecular structure

## Abstract

Our work in the area of synthesis of metal–organic frameworks (MOFs) based on organic *N*-oxides led to the crystallization of pyridine-4-carboxamidoxime *N*-oxide. Herein we report the first crystal structure of the title compound, C_6_H_7_N_3_O_2_ [systematic name: (*Z*)-4-(*N*′-hy­droxy­carbamimido­yl)pyridine *N*-oxide]. The hy­droxy­carbamimidoyl group is essentially coplanar with the aromatic ring, r.m.s.d. = 0.112 Å. The compound crystallizes in hydrogen-bonding layers built from the formation of strong O—H⋯O hydrogen bonds between the oxime oxygen atom and the oxygen atom of the *N*-oxide, and the formation of N—H⋯O hydrogen bonds between one amine nitro­gen atom and the *N*-oxide oxygen atom. These combined build *R* ^3^_4_(24) ring motifs in the crystal. The crystal structure has no π–π inter­actions.

## Abstract 2

The mol­ecular structure of pyridine-4-carboxamidoxime *N*-oxide is presented, which gives a two-dimensional supra­molecular crystal structure.

## Graphical abstract

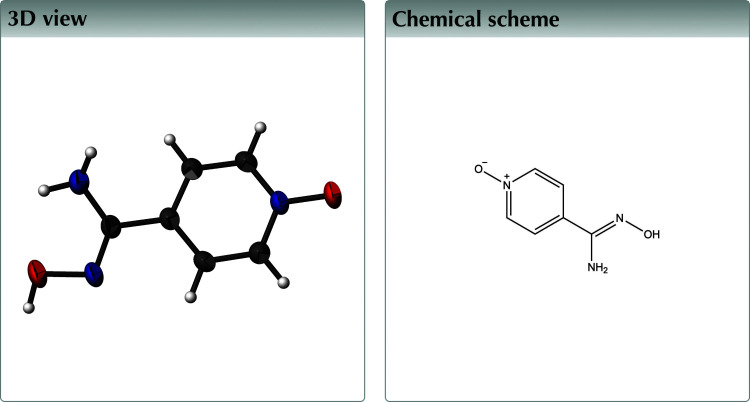

## Structure description

Since their first reported syntheses (Meisenheimer *et al.*, 1926 ▸), pyridine *N*-oxide and related compounds have garnered much inter­est in chemistry. We are particularly inter­ested in their uses in coordination polymers and as potential catalysts. The utility of these aromatic *N*-oxides to facilitate organic oxotransfer reactions has been well documented over the years (see, for example: Espenson, 2003 ▸). Many of these reactions are actually catalyzed by transition-metal inter­actions with the *N*-oxide ligands (see, for example: Moustafa *et al.*, 2014 ▸). Others have reported their use as coordination polymers (Ren *et al.*, 2018 ▸). We have also previously reported *N*-oxides used in coordination polymers of Mn (Kang *et al.*, 2017 ▸ and Lynch *et al.*, 2018 ▸). In this work, the syntheses of metal complexes of the title compound were attempted (Mn, Cu, Ce, Nd, Er, and Pr) by mixing the halide or nitrate salts of the metals with the title compound in methanol; unfortunately, all resulting crystals were of the uncomplexed ligand.

**Figure 1:**
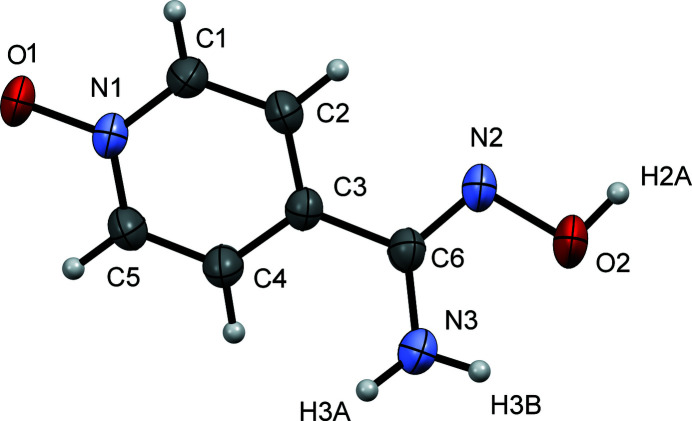
A view of the mol­ecular structure of the title compound, with the atom-labeling scheme. Displacement ellipsoids are drawn at the 50% probability level.

Herein we report the first crystal structure of pyridine-4-carboxamidoxime *N*-oxide (Fig. 1 ▸), which crystallizes in the monoclinic space group *P*2_1_/*c*. The mol­ecule is nearly planar with a r.m.s.d. of 0.112 Å for all non-hydrogen atoms, with the carbamimidoyl group slightly rotated by 15.09 (8)° with respect to the pyridine ring plane. N1—O1 has a distance of 1.3226 (18) Å and is consistent with normal *N*-oxide distances. The crystal structure contains a strong inter­molecular hydrogen bond between O2⋯O1^i^ which forms a chain running parallel to the *b* axis; the O2⋯O1^i^ separation is 2.6747 (19) Å. Another hydrogen bond is formed between N3⋯O1^ii^ which links neighboring chains together; the N3⋯O1^ii^ separation is 2.899 (2) Å [symmetry codes: (i) *x*, *y* + 1, *z*; (ii) *x*, −*y* + 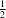 , *z* + 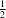 , see Table 1 ▸].

**Figure 2:**
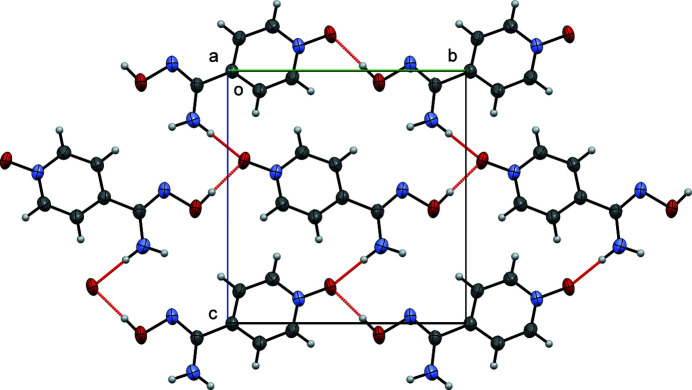
Crystal packing diagram of title compound viewed along [100]. Hydrogen bonds are colored red.

These hydrogen bonds link four mol­ecules together and form an 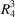 (24) ring motif in the crystal. Each mol­ecule is also part of four different *R*(24) synthons, generating sheets of hydrogen-bonding mol­ecules parallel to the (100) face of the unit cell (Fig. 2 ▸). There are no other short contacts or π–π inter­actions observed in the crystal.

## Synthesis and crystallization

An amount of 0.025 g of pyridine-4-carboxamidoxime *N*-oxide (Alfa Aesar) was weighed and dissolved in a 25 ml beaker in enough methanol to form a solution that allowed to slowly evaporate at room temperature. The clear crystals were analyzed on a Rigaku Xtal Miniflex.

## Refinement

Crystal data, data collection and structure refinement details are summarized in Table 2 ▸.

## Supplementary Material

Crystal structure: contains datablock(s) I. DOI: 10.1107/S2414314620013358/bh4056sup1.cif

Structure factors: contains datablock(s) I. DOI: 10.1107/S2414314620013358/bh4056Isup2.hkl

CCDC reference: 2035503

Additional supporting information:  crystallographic information; 3D view; checkCIF report

## Figures and Tables

**Table 1:** Hydrogen-bond geometry (Å, °)

*D*—H⋯*A*	*D*—H	H⋯*A*	*D*⋯*A*	*D*—H⋯*A*
O2—H2*A*⋯O1^i^	0.91 (3)	1.77 (3)	2.6747 (19)	172 (2)
N3—H3*A*⋯O1^ii^	0.91 (2)	2.00 (2)	2.899 (2)	167 (2)

Symmetry codes: (i) 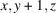 ; (ii) 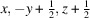 .

**Table 2:** Experimental details

Crystal data	
Chemical formula	C_6_H_7_N_3_O_2_
*M*_r_	153.15
Crystal system, space group	Monoclinic, *P*2_1_/*c*
Temperature (K)	170
*a*, *b*, *c* (Å)	7.4130 (8), 9.2858 (7), 10.1238 (10)
β (°)	102.841 (10)
*V* (Å^3^)	679.45 (11)
*Z*	4
Radiation type	Mo *K*α
μ (mm^−1^)	0.12
Crystal size (mm)	0.35 × 0.2 × 0.2

Data collection	
Diffractometer	Rigaku XtaLAB mini
Absorption correction	Multi-scan (*CrysAlis PRO*; Rigaku OD, 2018 ▸)
*T*_min_, *T*_max_	0.940, 1.000
No. of measured, independent and observed [*I* > 2σ(*I*)] reflections	5858, 1238, 961
*R*_int_	0.034
(sin θ/λ)_max_ (Å^−1^)	0.602

Refinement	
*R*[*F* ^2^ > 2σ(*F* ^2^)], *wR*(*F* ^2^), *S*	0.039, 0.101, 1.04
No. of reflections	1238
No. of parameters	113
No. of restraints	3
H-atom treatment	H atoms treated by a mixture of independent and constrained refinement
Δρ_max_, Δρ_min_ (e Å^−3^)	0.17, −0.15

Computer programs: *CrysAlis PRO* (Rigaku OD, 2018 ▸), *SHELXT* (Sheldrick, 2015*a* ▸), *SHELXL* (Sheldrick, 2015*b* ▸) and *OLEX2* (Dolomanov *et al.*, 2009 ▸).

## full crystallographic data

### Crystal data

**Table:**

C_6_H_7_N_3_O_2_	*F*(000) = 320
*M**_r_* = 153.15	*D*_x_ = 1.497 Mg m^−^^3^
Monoclinic, *P*2_1_/*c*	Mo *K*α radiation, λ = 0.71073 Å
*a* = 7.4130 (8) Å	Cell parameters from 3017 reflections
*b* = 9.2858 (7) Å	θ = 2.1–32.6°
*c* = 10.1238 (10) Å	µ = 0.12 mm^−^^1^
β = 102.841 (10)°	*T* = 170 K
*V* = 679.45 (11) Å^3^	Block, clear dark colourless
*Z* = 4	0.35 × 0.2 × 0.2 mm

### Data collection

**Table:**

Rigaku XtaLAB mini diffractometer	1238 independent reflections
Radiation source: fine-focus sealed X-ray tube, Enhance (Mo) X-ray Source	961 reflections with *I* > 2σ(*I*)
Graphite Monochromator monochromator	*R*_int_ = 0.034
Detector resolution: 13.6612 pixels mm^-1^	θ_max_ = 25.4°, θ_min_ = 2.8°
ω–scans	*h* = −8→8
Absorption correction: multi-scan (CrysAlisPro; Rigaku OD, 2018)	*k* = −11→11
*T*_min_ = 0.940, *T*_max_ = 1.000	*l* = −12→12
5858 measured reflections	

### Refinement

**Table:**

Refinement on *F*^2^	Secondary atom site location: difference Fourier map
Least-squares matrix: full	Hydrogen site location: mixed
*R*[*F*^2^ > 2σ(*F*^2^)] = 0.039	H atoms treated by a mixture of independent and constrained refinement
*wR*(*F*^2^) = 0.101	*w* = 1/[σ^2^(*F*_o_^2^) + (0.0443*P*)^2^ + 0.2172*P*] where *P* = (*F*_o_^2^ + 2*F*_c_^2^)/3
*S* = 1.04	(Δ/σ)_max_ < 0.001
1238 reflections	Δρ_max_ = 0.17 e Å^−^^3^
113 parameters	Δρ_min_ = −0.14 e Å^−^^3^
3 restraints	Extinction correction: SHELXL-2018/1 (Sheldrick 2015b), Fc^*^=kFc[1+0.001xFc^2^λ^3^/sin(2θ)]^-1/4^
Primary atom site location: dual	Extinction coefficient: 0.007 (2)

### Special details

**Table:**

Refinement. All carbon-bound H atoms were positioned geometrically and refined as riding, with C—H = 0.95 Å and *U*_iso_(H) = 1.2*U*_eq_(C). N—H and O—H hydrogen atoms were refined with free coordinates and isotropic displacement parameters.

### Fractional atomic coordinates and isotropic or equivalent isotropic displacement parameters (Å^2^)

**Table:**

	*x*	*y*	*z*	*U*_iso_*/*U*_eq_	
N1	0.7185 (2)	0.20253 (15)	0.40257 (15)	0.0361 (4)	
C1	0.6365 (3)	0.31255 (19)	0.32458 (18)	0.0392 (5)	
H1	0.565809	0.293556	0.235921	0.047*	
O1	0.6953 (2)	0.06920 (13)	0.35596 (13)	0.0498 (4)	
C2	0.6543 (2)	0.45181 (18)	0.37200 (17)	0.0371 (5)	
H2	0.596971	0.528186	0.315518	0.044*	
N2	0.7210 (2)	0.73345 (15)	0.47364 (16)	0.0434 (4)	
O2	0.7388 (2)	0.86472 (14)	0.54650 (15)	0.0628 (5)	
H2A	0.713 (3)	0.933 (3)	0.481 (2)	0.084 (8)*	
C3	0.7554 (2)	0.48156 (17)	0.50187 (16)	0.0311 (4)	
N3	0.8319 (3)	0.64526 (18)	0.69433 (16)	0.0450 (5)	
H3A	0.807 (3)	0.572 (2)	0.748 (2)	0.066 (7)*	
H3B	0.805 (3)	0.7337 (18)	0.722 (2)	0.059 (7)*	
C4	0.8403 (3)	0.36626 (19)	0.57809 (18)	0.0382 (5)	
H4	0.912748	0.382572	0.666750	0.046*	
C5	0.8210 (3)	0.22887 (19)	0.52697 (19)	0.0407 (5)	
H5	0.881159	0.151356	0.580441	0.049*	
C6	0.7673 (2)	0.62923 (18)	0.55763 (17)	0.0337 (4)	

### Atomic displacement parameters (Å^2^)

**Table:**

	*U*^11^	*U*^22^	*U*^33^	*U*^12^	*U*^13^	*U*^23^
N1	0.0481 (9)	0.0226 (7)	0.0376 (8)	0.0000 (7)	0.0092 (7)	−0.0035 (6)
C1	0.0496 (11)	0.0309 (10)	0.0334 (9)	0.0011 (8)	0.0016 (8)	−0.0018 (8)
O1	0.0790 (10)	0.0213 (7)	0.0470 (8)	−0.0002 (6)	0.0098 (7)	−0.0077 (6)
C2	0.0468 (11)	0.0263 (9)	0.0359 (10)	0.0036 (8)	0.0044 (8)	0.0038 (7)
N2	0.0670 (11)	0.0213 (8)	0.0419 (9)	−0.0020 (7)	0.0119 (8)	−0.0021 (7)
O2	0.1140 (14)	0.0213 (7)	0.0520 (9)	−0.0023 (8)	0.0158 (9)	−0.0038 (7)
C3	0.0334 (9)	0.0255 (9)	0.0348 (9)	−0.0015 (7)	0.0083 (8)	−0.0008 (7)
N3	0.0656 (11)	0.0286 (9)	0.0387 (9)	−0.0060 (8)	0.0068 (8)	−0.0042 (7)
C4	0.0446 (11)	0.0299 (9)	0.0360 (10)	0.0018 (8)	0.0002 (8)	−0.0005 (8)
C5	0.0517 (11)	0.0288 (10)	0.0377 (10)	0.0064 (8)	0.0018 (9)	0.0036 (8)
C6	0.0382 (10)	0.0265 (9)	0.0365 (10)	−0.0046 (7)	0.0088 (8)	−0.0013 (8)

### Geometric parameters (Å, º)

**Table:**

N1—C1	1.350 (2)	O2—H2A	0.91 (3)
N1—O1	1.3226 (18)	C3—C4	1.386 (2)
N1—C5	1.341 (2)	C3—C6	1.478 (2)
C1—H1	0.9500	N3—H3A	0.912 (16)
C1—C2	1.376 (2)	N3—H3B	0.903 (15)
C2—H2	0.9500	N3—C6	1.368 (2)
C2—C3	1.389 (2)	C4—H4	0.9500
N2—O2	1.4156 (19)	C4—C5	1.372 (2)
N2—C6	1.284 (2)	C5—H5	0.9500
			
O1—N1—C1	119.59 (15)	C4—C3—C6	121.51 (15)
O1—N1—C5	120.50 (15)	H3A—N3—H3B	114 (2)
C5—N1—C1	119.91 (15)	C6—N3—H3A	116.5 (14)
N1—C1—H1	119.6	C6—N3—H3B	111.1 (14)
N1—C1—C2	120.77 (16)	C3—C4—H4	119.6
C2—C1—H1	119.6	C5—C4—C3	120.77 (16)
C1—C2—H2	119.8	C5—C4—H4	119.6
C1—C2—C3	120.47 (16)	N1—C5—C4	120.90 (16)
C3—C2—H2	119.8	N1—C5—H5	119.5
C6—N2—O2	108.89 (15)	C4—C5—H5	119.5
N2—O2—H2A	103.8 (16)	N2—C6—C3	117.52 (15)
C2—C3—C6	121.33 (15)	N2—C6—N3	124.75 (16)
C4—C3—C2	117.14 (16)	N3—C6—C3	117.71 (15)
			
N1—C1—C2—C3	−0.7 (3)	C2—C3—C6—N3	165.27 (17)
C1—N1—C5—C4	1.7 (3)	O2—N2—C6—C3	179.01 (15)
C1—C2—C3—C4	1.8 (3)	O2—N2—C6—N3	−3.0 (3)
C1—C2—C3—C6	−176.45 (16)	C3—C4—C5—N1	−0.5 (3)
O1—N1—C1—C2	178.21 (17)	C4—C3—C6—N2	165.18 (17)
O1—N1—C5—C4	−177.58 (17)	C4—C3—C6—N3	−13.0 (3)
C2—C3—C4—C5	−1.2 (3)	C5—N1—C1—C2	−1.1 (3)
C2—C3—C6—N2	−16.6 (3)	C6—C3—C4—C5	177.05 (17)

### Hydrogen-bond geometry (Å, º)

**Table:**

*D*—H···*A*	*D*—H	H···*A*	*D*···*A*	*D*—H···*A*
O2—H2*A*···O1^i^	0.91 (3)	1.77 (3)	2.6747 (19)	172 (2)
N3—H3*A*···O1^ii^	0.91 (2)	2.00 (2)	2.899 (2)	167 (2)

Symmetry codes: (i) *x*, *y*+1, *z*; (ii) *x*, −*y*+1/2, *z*+1/2.
